# Influence of Printing Orientation on the Mechanical Properties of Provisional Polymeric Materials Produced by 3D Printing

**DOI:** 10.3390/polym17030265

**Published:** 2025-01-21

**Authors:** Fábio Hideo Kaiahara, Eliane Cristina Gava Pizi, Fabiana Gouveia Straioto, Lucas David Galvani, Milton Carlos Kuga, Thalita Ayres Arrué, Ageu Raupp Junior, Marcus Vinícius Reis Só, Jefferson Ricardo Pereira, Hugo Vidotti

**Affiliations:** 1 University of Western São Paulo, Presidente Prudente 19050-680, Brazil; fhkaiahara@yahoo.com.br (F.H.K.); elianepizi@unoeste.br (E.C.G.P.); fabianagouveia@yahoo.com.br (F.G.S.); 2 State University of São Paulo, UNESP, São Paulo 18618687, Brazil; lucas.galvani@icloud.com (L.D.G.); milton.kuga@unesp.br (M.C.K.); 3 Federal University of Rio Grande do Sul—UFRGS, Porto Alegre 91540-000, Brazil; thalita.arrue@hotmail.com (T.A.A.); endo-so@hotmail.com (M.V.R.S.); 4 University Southern Santa Catarina, Tubarão 88704-900, Brazil; ageurauppjr@hotmail.com; 5 Independent Researcher, Presidente Prudente 19050-680, Brazil; havidotti@yahoo.com.br

**Keywords:** denture, partial, temporary, printing, three-dimensional, computer-aided design, resin cements

## Abstract

This study investigates the impact of printing layer orientation on the mechanical properties of 3D-printed temporary prosthetic materials. Traditionally, temporary prostheses are fabricated using acrylic resin (polymethyl methacrylate), but advancements have introduced bis-acrylic resins, CAD/CAM-based acrylic resin (milled), and 3D printing technologies. In 3D printing, material is manufactured in overlapping layers, which can be oriented in different directions, directly affecting the material’s resistance. Specimens were designed as bars (2 mm × 2 mm × 25 mm) and grouped according to their printing orientation: BP0 (0 degrees), BP45 (45 degrees), and BP90 (90 degrees). The models were created using Fusion 360 software (version 2.0.12600) and printed on a 3D DLP printer with DLP Slicer software (Chitu DLP Slicer, CBD Tech, version v1.9.0). The bars were then subjected to 3-point bending tests using an Instron Universal Testing Machine to measure Flexural Strength (FS) and Flexural Modulus (FM). Results demonstrated that the BP90 group exhibited the highest Flexural Strength (114.71 ± 7.61 MPa), followed by BP45 (90.10 ± 8.45 MPa) and BP0 (80.90 ± 4.01 MPa). Flexural Modulus was also highest in the BP90 group (3.74 ± 3.64 GPa), followed by BP45 (2.85 ± 2.70 GPa) and BP0 (2.52 ± 2.44 GPa). Significant statistical differences (*p* < 0.05) were observed, indicating changes in the mechanical properties of the 3D-printed material. The study concludes that printing orientation significantly influences the mechanical properties of temporary prosthetic materials, making the selection of an optimal orientation essential to enhance material performance for its intended application.

## 1. Introduction

Temporary prostheses are vital in dental rehabilitation, providing pulp protection, safeguarding periodontal health, restoring aesthetics, and reestablishing chewing function. These protheses help preserve existing teeth and enhance patient self-esteem [1]. Traditionally, they were made from self-polymerizing acrylic resins [2]. However, this method has several drawbacks, including long production time, risk of fracture during long-term use, biofilm accumulation due to surface changes, distortion in the impression materials, distortion in the model material, and improper technique [2,3,4,5,6].

The introduction of digital techniques, specifically computer-aided design and manufacturing (CAD/CAM), has revolutionized prosthetic production in recent years [1,6,7]. CAD/CAM uses an intraoral scanner to generate precise 3D models of the oral cavity, facilitating the rapid creation of prosthetic components [3,6,8,9]. Although the technology offers high precision, factors such as saliva, patient movement, and limited intraoral space can affect accuracy [4,8].

3D printing, another breakthrough, involves the additive layer-by-layer manufacturing of objects [10,11], which offers various printing methods, including Fused Deposition Modeling (FDM), Digital Light Processing (DLP), and Stereolithography (SLA). DLP, in particular, stands out for its speed and accuracy, making it suitable for dental applications. This method enables the creation of highly detailed and robust structures, addressing the limitations of traditional prosthesis production by eliminating the need for impression materials, plaster molds, and manual processing [12,13].

The 3D printing process allows for the rapid and precise production of temporary restorations, improving both efficiency and material quality. Advances in 4D printing, which involves materials that respond to external stimuli, are also on the horizon, potentially allowing for dental devices that adapt to changing oral conditions over time [13].

Arif ZU et al. [14] compared Digital Light Processing (DLP) with other 3D printing methods, highlighting its speed and precision, particularly in hydrogel curing. Unlike Stereolithography (SLA), which cures layer by layer, DLP cures entire layers simultaneously, making it more efficient for complex structures. Fused Deposition Modeling (FDM) struggles with heat-sensitive materials like hydrogels, while DLP’s versatility allows for the creation of hydrogels with adaptive properties, making it especially useful in dental applications.

Computer-aided manufacturing (CAD/CAM) overcomes the limitations of traditional temporary prosthesis production by automating the process and improving efficiency. It streamlines treatment planning, thereby reducing appointments, enhances accuracy and fit, minimizes distortion, and cuts material waste, benefiting both dental professionals and patients [9,15,16,17].

In addition to reducing production time and improving the fit and longevity of prostheses, CAD/CAM and 3D printing technologies reduce material waste and improve overall treatment efficiency. These advancements offer a significant advantage over conventional methods, but there remains a need for further research, particularly in understanding how printing parameters like layer orientation influence the mechanical properties of materials. Therefore, the present study aims to evaluate the influence of printing layer orientation on the mechanical property (flexural strength) of temporary prosthetic materials.

## 2. Materials and Methods

### 2.1. Groups and Specimens Preparation

**Figure 1:**
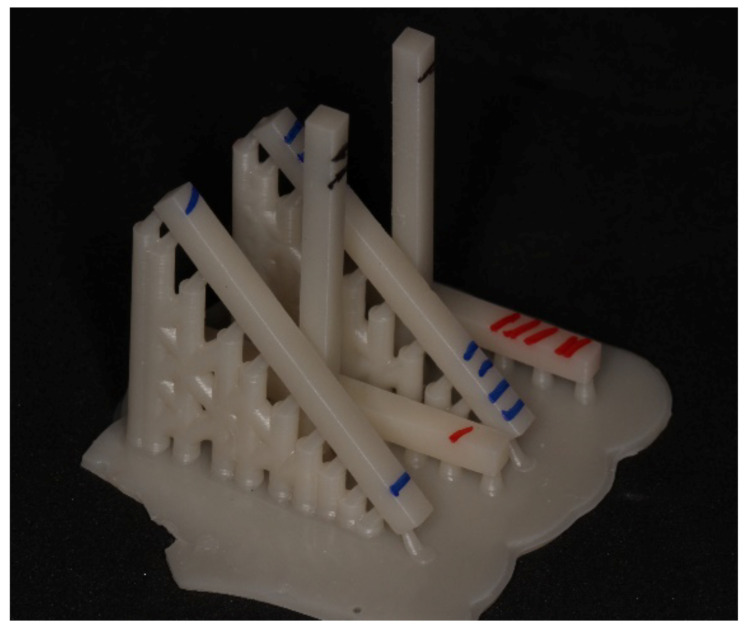
Specimens removed and identified (red P0, blue P45, and black P90).

This study involved an experimental group with varying 3D printing orientations for provisional resin and a control group (milled provisional resin). The experimental group was divided into three subgroups, P0, P45, and P90, with bar-shaped specimens created for each subgroup. The specimens were designed using CAD software (Fusion 360, Autodesk, version 2.0.12600). The tray-forming software (Chitu DLP Slicer, CBD Tech, version v1.9.0) allowed for the orientation of experimental subgroup models at different angles (0, 45, and 90 degrees) relative to the printing tray, with layer thickness standardized in micrometers along the *Z*-axis (Table 1). After finalizing the design and setting the angles, the specimens were printed on a 3D DLP printer (BASIC PRINTER X, 3DBasic, Marília, São Paulo, Brazil). A single batch of a specific resin (COSMOS TEMP, A1, Yller Biomaterials, Pelotas, Brazil) was used for 3D printing of the dental provisionals. This resin, formulated for temporary restorations, consists of an organic matrix based on methacrylates like Bis-GMA and TEGDMA, along with inorganic fillers such as silica, providing mechanical strength and suitable aesthetics for provisional use. The light-curing system, activated by blue light (approximately 470 nm), enables fast and controlled polymerization, resulting in excellent marginal adaptation as well as ease in finishing and polishing. After printing, the specimens were removed from the forming base and identified by group (red for P0, blue for P45, and black for P90) (Figure 1).

### 2.2. 3D Printing Setting

**Figure 2:**
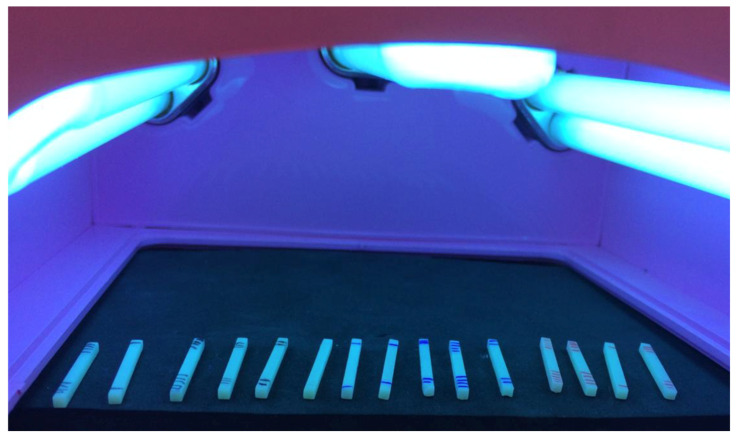
Specimens under polymerization process (series of 8 exposures of 30 s polymerization).

Supports for the 0°, 45°, and 90° groups were manually inserted, maintaining a standardized 2 mm distance between each support. The 3D printing process was conducted with a layer exposure time of 5.5 s and a layer thickness of 50 microns. After printing, the specimens were removed from the printing platform and washed in isopropyl alcohol in two separate baths, each lasting two minutes. The specimens were then dried and post-cured in a curing unit for 10 min. Dimensional measurements were taken using a digital caliper with a precision of 0.01 mm (Mitutoyo, Kawasaki, Japan), and the specimens were identified for final polymerization (post-curing) using the Anycubic Wash and Cure unit. A series of 8 exposures (2 per face) were applied for polymerization, with each exposure lasting 30 s, totaling 60 s per face of the bar (Figure 2).

### 2.3. Conventional Milled Resin

**Figure 3:**
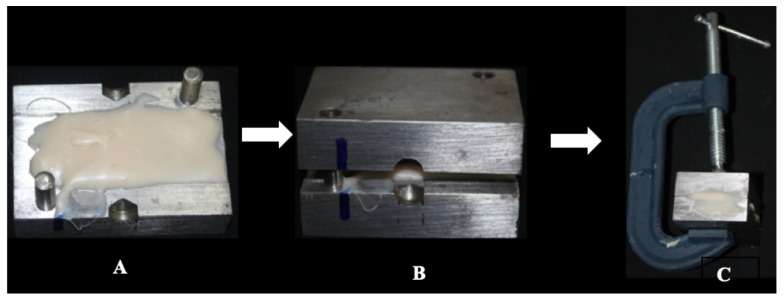
2-parts metallic matrix (**A**), joined with type C clamp (**B**,**C**).

In the control group, bars measuring 2 mm × 2 mm × 25 mm (*n* = 15) were fabricated using polymethyl methacrylate (PMMA) (Duralay Color 81—Reliance Dental MFG Co., Ltd., Itasca, IL, USA), following ISO 10477 standards [18]. A powder-to-monomer ratio of 20 g of powder to 10 mL of monomer was prepared, as specified by the manufacturer, in a glass paladon jar with a lid. The preparation commenced by mixing the powder with the liquid using a spatula (No. 36) until the acrylic resin reached its plastic phase. At this point, the mixture was placed in the center of a two-part metallic matrix, with one part being flat and the other featuring a 2 mm depression. Both parts were secured with a type C clamp to ensure complete closure (Figure 3). The assembly was then placed in a bubble-elimination pan (Essence Dental VH, Schaan, Liechtenstein) under a pressure of 25 pounds until the resin underwent final polymerization, resulting in uniform acrylic resin plates.

**Figure 4:**
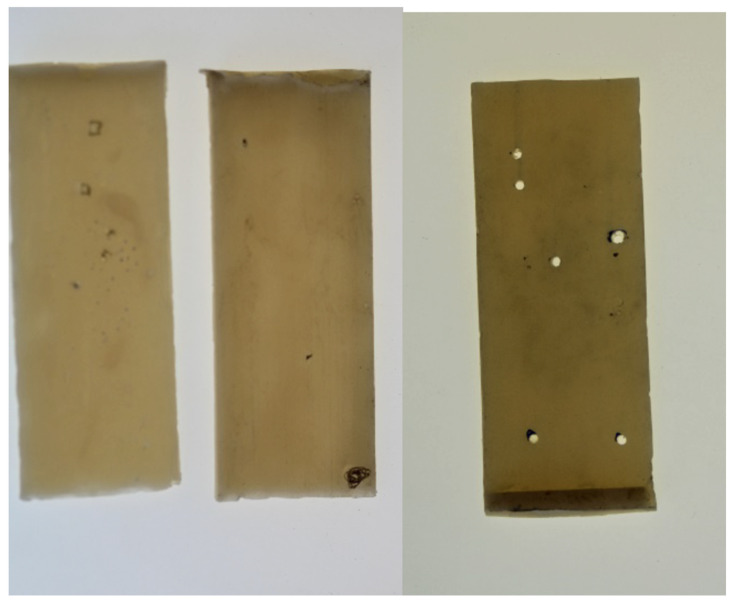
PMMA plates with possible bubbles marks and perforations.

Subsequently, the plates from the control group were analyzed under light, and marks were made on any visible bubbles on their surfaces. Perforations were created at the locations of the bubbles to establish exclusion criteria for the specimens (Figure 4).

**Figure 5:**
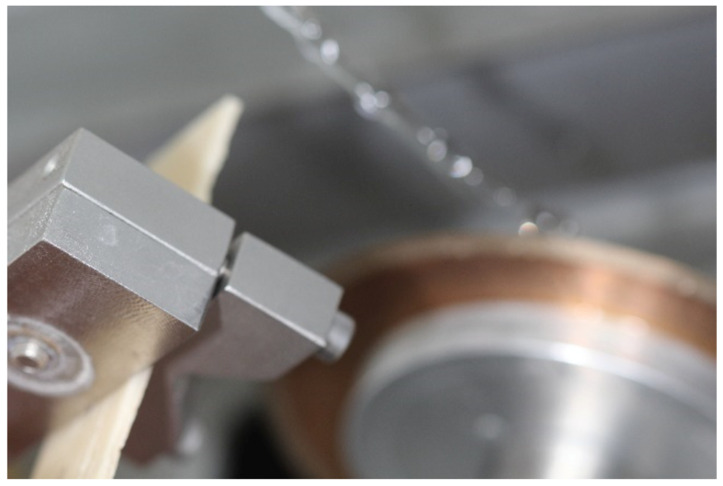
Acrylic resin plates base-fixed and sectioned in a precision cutting machine.

The manufactured plates were fixed to the base and sectioned using a precision cutting machine (LCD 1200 rpm Metallographic Cutter, Bueler, Lake Bluff, IL, USA) to a thickness of 2 mm (Figure 5), resulting in 25 bars with dimensions identical to those of the control group, obtained from the acrylic resin plates. Following visual analysis with a magnifying glass, 15 bars were selected for testing. Bars exhibiting bubbles or imperfections were excluded from the study. The freshly cut surfaces of the bars were polished with #1200 sandpaper to standardize the roughness of all faces. Subsequently, the bars were stored in water at a temperature of 36 °C for 24 h prior to the three-point bending tests. The dimensions of the specimens were measured individually using a digital caliper. Each specimen was numbered and recorded in a spreadsheet to facilitate the subsequent three-point flexural strength testing.

### 2.4. Three-Point Bending Test

**Figure 6:**
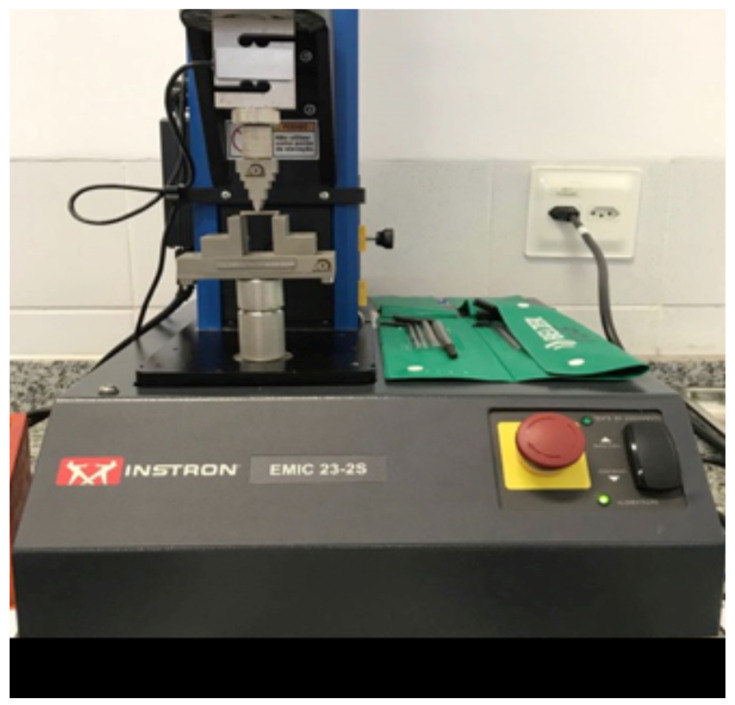
Emic/Instron 23-2S.

**Figure 7:**
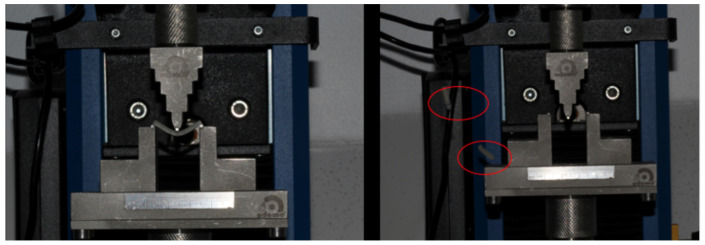
3-point bending test.

After storage in water, the specimens from the immediate subgroup were subjected to the three-point bending test using a mechanical testing machine (Instron 23-2S, Norwood, MA, USA) connected to a computer. The distance between the lower supports was set at 20 mm, and the testing speed was maintained at 1 mm/min with a load cell of 100 Kgf (Figure 6 and Figure 7). The three-point bending test was conducted to evaluate the flexural strength (FS) and calculate the flexural modulus (FM). The flexural strength tests adhered to the ISO 10477 standard (Standardization IOF. ISO 10477 Dentistry Polymer-Based Crown and Bridge Materials, 2004) [18]. The tests were performed at a room temperature of 22 °C, with calibration conducted using the machine’s software (Bluehill Universal 4).

FR = 3Fl/2bh^2^

FM = F_l_^3^/4bh^3^d

FR represents the flexural strength measured in MPa, while FM denotes the flexural modulus expressed in GPa. In these equations, F refers to the maximum force applied in Newtons (N); l is the distance between the lower supports; b is the width; and h is the height of the specimen (in mm). The variable m signifies the slope of the most linear portion of the force-displacement curve. The variable “d” corresponds to the area under the force-displacement curve, representing the energy applied during deflection until the specimen fractures, measured in Joules (J). F1 indicates the fracture work in kJ/m^2^. Microsoft Excel (Version 16.89.1, Microsoft) was used for these calculations.

The data were subjected to statistical analysis of Variance by 2 Criteria (two-way ANOVA) and Tukey’s post-test for multiple comparisons.

## 3. Results

The observed results indicated a statistical difference between the groups regarding resistance values and flexural modulus (*p* < 0.05). Comparisons revealed that the P90 group exhibited significantly higher values of flexural strength (114.71 ± 7.61 MPa). Similarly, the P90 group showed significantly higher values for the flexural modulus (*p* < 0.05). The mean and standard deviation values for resistance and flexural modulus are presented in Table 2.

## 4. Discussion

Studying the biomechanical behavior of materials used in temporary restorations that can be integrated into clinical practice, along with technological advancements, is essential for enabling agility and predictability in rehabilitation, from digital planning to 3D printing of temporary prostheses. The findings of the present study indicate that the printing angle significantly affects the mechanical properties of materials used in temporary prostheses.

The biomechanical properties, including resistance and flexural modulus (Table 2), exhibited higher values when the printing inclination was adjusted to 45 and 90 degrees compared to traditional acrylic resin. These data support the notion that this method of manufacturing temporary prostheses can serve as an alternative solution to the frequent problem of fractures in temporary prostheses, particularly in extensive or long-term rehabilitations.

Despite the limited literature on this topic, discussions surrounding the results of this study are warranted. Published data indicate isotropy concerning the printing direction. In terms of a material’s resistance to fracture, the optimal printing direction for an occlusal device is vertical [19].

It is crucial to evaluate other materials with varying compositions. During the printing process, voids may form between layers due to the mechanics of the procedure. Specifically, when the printing platform separates from the bottom of the tank, a negative pressure is generated, allowing air to infiltrate the resin. The elimination of dissolved voids has been shown to depend on resin viscosity, decreasing as viscosity increases; therefore, using a low-viscosity resin could enhance its mechanical properties [20].

Factors related to biocompatibility must also be considered when selecting provisional restorative materials, as they will remain in the oral cavity during the period that final pieces are being manufactured. A study [12] evaluated the cytocompatibility of dental materials for provisional restorations fabricated using DLP-type 3D printing and self-curing technologies, focusing on cell adhesion, morphology, and proliferation. Fibroblasts were surrounded by tissue cells, and an increase in cell adhesion and well-extended filopodia was observed in specimens manufactured via 3D printing. Comparatively, 3D printed specimens demonstrated superior biocompatibility relative to self-polymerizing resins [21]. The determination of the printing angle not only influenced resistance and flexural modulus but also affected bacterial colonization. According to a study by Shim et al. [22], the highest proportion of *Candida albicans* was observed on surfaces printed with an orientation of 0 degrees, followed by 45 and 90 degrees, with statistical significance. This orientation also influenced printing accuracy, flexural strength, and roughness [23].

Among the limitations of the study, the use of only one commercial brand of printing resin and the evaluation of biomechanical characteristics through resistance and flexural modulus analysis are noteworthy. While this study assessed printing angulation, Dizon et al. determined that layer thickness has a greater impact on object strength than printing direction [24].

To comprehend the clinical implications of this prosthetic manufacturing method, it is also essential to understand the wear behavior of these materials. A critical factor for medium-term use is the high wear resistance of the materials, which is necessary to maintain vertical dimension [25], color stability, and marginal adaptation [21].

The printing inclination may vary based on the type of material and the final product to be printed. Based on the results of the present study, 3D printing at a 90-degree angle is recommended for printing temporary prostheses, as the mechanical properties could be improved, thus enhancing the prognosis. However, it is important to note that printing at this angle will also increase printing time. Despite the growing demand for these high-tech restorations, additional information regarding the chemical composition and mechanical properties of these new temporary printed materials is necessary. Further studies should investigate layer thickness of specimens and different brands of 3D printing resins to ensure that printing orientation truly influences the mechanical properties of these materials.

## 5. Conclusions

The studied groups demonstrated statistically significant differences, indicating that the mechanical properties of this material are enhanced when printed at a 90-degree orientation. This finding suggests that the choice of printing orientation can significantly influence the material’s applicability, depending on the specific characteristics and functional requirements of the printed object. To improve the mechanical characteristics of 3D printing materials, we can suggest using the 90 degree orientation despite the increased working time. Other studies with different materials should be conducted to guarantee that this is a common characteristic among all the materials.

## Figures and Tables

**Table 1:** Study groups and studied materials.

Group	Material	Impression Angulation
Control	PMMA, Duralay color 81—Reliance Dental MGF Co., Ltd., Itasca, IL, USA	-
P0	COSMOS TEMP, color A1, Yller	0 degree
P45	COSMOS TEMP, color A1, Yller	45 degrees
P90	COSMOS TEMP, color A1, Yller	90 degrees

**Table 2:** Data obtained from the mechanical test of the resistance and flexural modulus values (mean, ±standard deviation) of the tested groups (*n* = 5).

Groups	Flexural Strength (MPa)	Flexural Modulus (MPa)
Control	78.13 ± 7.94 ^a^	2.80 ± 2.77 ^a^
P0	80.90 ± 4.0 ^ab^	2.52 ± 2.44 ^ab^
P45	90.10 ± 8.45 ^b^	2.85 ± 2.70 ^b^
P90	114.71 ± 7.61 ^c^	3.74 ± 3.64 ^c^

Different letters analyzed in column indicate statistically significant differences between groups (Anova, Tukey’s Post Test, *p* < 0.05).

## Data Availability

Data are contained within the article.
